# Real-time imaging of cGMP signaling shows pronounced differences between glomerular endothelial cells and podocytes

**DOI:** 10.1038/s41598-024-76768-1

**Published:** 2024-10-30

**Authors:** Nelli Rutkowski, Frederik Görlitz, Eva Wiesner, Julia Binz-Lotter, Susanne Feil, Robert Feil, Thomas Benzing, Matthias J. Hackl

**Affiliations:** 1 Department II Internal Medicine and Center for Molecular Medicine Cologne, University of Cologne, Faculty of Medicine and University Hospital Cologne, Cologne, Germany; 2 Cluster of Excellence Cellular Stress Responses in Aging- associated Diseases (CECAD), University of Cologne, Faculty of Medicine and University Hospital Cologne, Cologne, Germany; 3 Bio- and Nanophotonics, Department of Microsystem Engineering, University of Freiburg, Freiburg, Germany; 4 Interfakultäres Institut für Biochemie (IFIB), University of Tübingen, Tübingen, Germany; 5 Nephrolab Cologne, CECAD Research Center, University Hospital of Cologne, Joseph-Stelzmann-Str. 26, 50931 Cologne, Germany

**Keywords:** cGMP signaling, sGC, pGC, Podocyte, Glomerular endothelial cell, GECs, cGi500, FRET, Podocytes, Glomerulus, Fluorescence imaging, Microscopy, Nephrology, Kidney

## Abstract

Recent clinical trials of drugs enhancing cyclic guanosine monophosphate (cGMP) signaling for cardiovascular diseases have renewed interest in cGMP biology within the kidney. However, the role of cGMP signaling in glomerular endothelial cells (GECs) and podocytes remains largely unexplored. Using acute kidney slices from mice expressing the FRET-based cGMP biosensor cGi500 in endothelial cells or podocytes enabled real-time visualization of cGMP. Stimulation with atrial natriuretic peptide (ANP) or SNAP (NO donor) and various phosphodiesterase (PDE) inhibitors elevated intracellular cGMP in both cell types. GECs showed a transient cGMP response upon particulate or soluble guanylyl cyclase activation, while the cGMP response in podocytes reached a plateau following ANP administration. Co-stimulation (ANP + SNAP) led to an additive response in GECs. The administration of PDE inhibitors revealed a broader basal PDE activity in GECs dominated by PDE2a. In podocytes, basal PDE activity was mainly restricted to PDE3 and PDE5 activity. Our data demonstrate the existence of both guanylyl cyclase pathways in GECs and podocytes with cell-specific differences in cGMP synthesis and degradation, potentially suggesting new therapeutic options for kidney diseases.

**Supplementary Information:**

The online version contains supplementary material available at 10.1038/s41598-024-76768-1.

## Introduction

Recent clinical trials have demonstrated renoprotective effects of enhancing cGMP signaling in patients^1,2^, and ongoing studies are investigating further substances showing renoprotective effects in animal models^3,4^. The second messenger cGMP mediates signal transduction by activating downstream effector molecules such as protein kinase G (PKG), cyclic nucleotide-gated (CNG) channels or phosphodiesterases (PDEs), which degrade cGMP. Two types of guanylyl cyclases catalyze the conversion of guanosine-5’-triphosphate (GTP) to cGMP. These are particulate/membrane-bound receptors with an intracellular guanylyl cyclase domain (pGC-A/B/C) and soluble guanylyl cyclases (sGC) located in the cytosol^5^. Natriuretic peptides, such as atrial natriuretic peptide (ANP), activate the particulate guanylyl cyclase (pGC) signaling cascade via ligand-receptor binding, causing natriuresis, diuresis, suppression of the renin-angiotensin-aldosterone system (RAAS), and an increase in the glomerular filtration rate (GFR)^6–8^. NO/sGC-mediated cGMP synthesis involves nitric oxide synthases (NOS), which generate NO through the conversion of L-arginine to L-citrulline. NO diffuses across cell membranes and activates sGC present in target cells^5^. The NO/sGC/cGMP pathway influences glomerular function by dilating both pre- and post-glomerular arterioles and counteracts the effects of reactive oxygen species (ROS)^9–11^. Several studies have shown that cGMP levels vary across different compartments within the same cell, due to the independent regulation of cGMP synthesis by sGC or pGC and its degradation by various PDE isoforms^12–20^, underlining the need to study mechanisms of cGMP synthesis and degradation in each cell.

In disease states, cGMP signaling is diminished, suggesting a therapeutic benefit of its restoration. Several approved drugs elevating cGMP signaling became available in recent years, targeting key enzymes of the cGMP pathways^21,22^. Sacubitril inhibits the degradation of ANP^23,24^, whereas riociguat and vericiguat activate sGC, and sildenafil and tadalafil inhibit PDE5^25–28^. Studies using animal models aimed to enhance cGMP signaling have demonstrated a reduction of kidney injury in diabetes and chronic kidney disease^3,4,29,30^. Additionally, these models have shown potential therapeutic benefits in other cardio-renal pathologies^24,27,31^, thereby renewing the interest of nephrologists in the role of cGMP signaling.

To date, the role of GECs and podocytes in the renoprotective effect of cGMP signaling remains unclear. Measurements of cGMP levels in podocytes mostly rely on cell culture work, utilizing conventional biochemical techniques such as immunohistochemistry, radioimmunoassays (RIAs), and enzyme-linked immunoassays (ELISAs)^31–35^. However, these techniques require cell lysis and often involve the addition of unspecific PDE inhibitors to enhance sensitivity. This inhibition disrupts cGMP dynamics and precludes a detailed analysis of signaling processes. The development of Förster resonance energy transfer (FRET)-based cGMP biosensors, such as cGi500^36,37^, enables real-time monitoring of cGMP fluctuations within living cells. Binding of cGMP leads to a conformational change of the indicator protein cGi500, resulting in increased CFP fluorescence (donor) and decreased YFP fluorescence (acceptor). This sensor is capable of visualizing cGMP changes in intact tissue in high spatial and temporal resolution, overcoming some of the hurdles that have hampered cGMP research in previous years^15,38,39^.

The existence of each cGMP signaling pathway is differentially debated for GECs and podocytes^31,32,34,40–42^. This led us to the hypothesis, that cGMP dynamics in glomerular cell types are fundamentally different. Therefore, we utilized the cGMP biosensor cGi500 with cell-specific expression for live cell imaging of mouse kidney tissue. Cell-specific data for each cGMP signaling pathway allowed a comparative analysis between GECs and podocytes to further elucidate differences in cGMP synthesis and degradation.

## Methods

### In vitro characterization of cGi500

The plasmid DNA MH20_pCMV_cGi500 (7767 bp, Supplemental Fig. 1) encoding the single-chain cGMP biosensor cGi500 was provided by the Feil lab based on the sequence designed^37^ by Michael Russwurm.

HEK 293T cells maintained in DMEM (Thermo Fisher Scientific, 31966-021) supplemented with 10% FBS (Thermo Fisher Scientific, 10270106) were transiently transfected by calcium-phosphate mediated DNA transfer. Cells were incubated at 37 °C with replacement of the present medium after 7 h with phenol red-free DMEM (Sigma-Aldrich, D1145) supplemented with 10% FBS, 1% GlutaMAX™ (Thermo Fisher Scientific, 35050-061) and 1% sodium pyruvate solution (Sigma-Aldrich, S8636). Transfected cells from a 10-cm dish (1 × 10^7^ cells) were harvested and lysed according to a previously published protocol^37^, with minor modifications. Considering temperature and light sensitivity, all preparation steps were performed on ice with the avoidance of strong light exposure. The cells were washed twice with 5 ml of ice-cold sterile PBS, followed by 300 µl of freshly prepared ice-cold homogenization buffer (1 mM EDTA, 50 mM NaCl, 50 mM triethanolamine/HCl, pH 7.4, 2 mM dithiothreitol, 100-fold dilution of a protease-inhibitor cocktail (Roche, 4693132001)). The adherent cells were scraped off, and the lysate was transferred to sterile tubes. A cytosolic fraction was obtained by sonication (5 s ON, 30 s OFF, 1 cycle), followed by ultracentrifugation at 100,000 g for 30 min at 4 °C (Optima MAX-XP, Beckman Coulter, Rotor TLA-55). The volume for 25 µg protein (BCA assay, Thermo Fisher Scientific, 23225) was added to freshly prepared buffer A (50 mM triethanolamine/HCl, pH 7.4, 2 mM dithiothreitol, 10 mM MgCl_2_)) to achieve a final volume of 300 µl. An ascending cGMP (Sigma-Aldrich, G7504) dilution series (0.01 µM to 100 µM) was added (2.1 µl) and incubated for 5 min. The fluorescence of the biosensor was recorded using a confocal microscope (LSM Meta 710 Axio Observer, Zeiss) equipped with an EC Plan-Neofluar 10x/0.3 objective and ZEN 2009 software (Zeiss). Intensity changes in the 512 × 512 × 16-bit emission images (mean fluorescence intensity) were analyzed using ImageJ (NIH) to calculate the CFP/YFP emission ratio (FRET ratio). The performed concentration-response curve analysis reflects the concentration-dependent cGMP binding properties of the sensor.

### Mouse lines

Cell-specific cytosolic expression of the FRET-based biosensor cGi500 was accomplished by crossing the ‘floxed’ cGi500 sensor line^36^ Gt(ROSA)26Sor^tm1(CAG−ECFP/EYFP*)Feil^(mixed background 129 SV, C57BL/6 N, (C57BL6/NRj > 70%)) with Pod:Cre mice^43^ to achieve sensor expression in podocytes, and Tie2:Cre mice^44^ or Cdh5:Cre mice^45^ for expression in GECs. Experiments with Pod:Cre/cGi500 mice were performed with homozygous cGi500 alleles. Experiments with Tie2:Cre/cGi500 mice were performed with the first filial generation of offspring (carrying only one cGi500 allele) due to reported nonspecific recombination in the F2 generation^46,47^. Experiments with Cdh5:Cre mice were also conducted using the first-generation of offspring.

Mice of both sexes, aged 8–16 weeks, were used. Animals were housed under standardized, specific pathogen-free conditions and kept in individually ventilated cage systems (IVC cages type II long) with centrally monitored light periodicity (light/dark cycle 12 h/12 h, 55 ± 10% humidity, 22 ± 2 °C room temperature). Experimental procedures were evaluated and approved by the Landesamt für Natur, Umwelt und Verbraucherschutz (LANUV) NRW (VSG 81-02.04.2018.A351.). Animal experiments were conducted in accordance with the ARRIVE guidelines.

### Poly-L-lysine coating

To immobilize kidney slices for real-time imaging, coverslips (∅ 12 mm, #1.5, Gerhard Menzel) were coated with approximately 700 µl of a 0.1% poly-L-lysine solution (Sigma-Aldrich, P8920) for 1 h at RT.

### Acute kidney slices (AKS)

Kidney slices were prepared according to the protocol of Poosti et al.^48^, with the following modifications: On the day of the experiment, 1 L of 1X Krebs-Henseleit buffer (KHB) was prepared, kept cold, and gassed with 95% O_2_/5% CO_2_ for 30 min using a gas dispersion tube with a fritted disc. Subsequently, the solution was titrated to achieve a pH of 7.42. Mice were euthanized by cervical dislocation, and the kidneys were directly transferred to ice-cold 1X KHB, followed by removal of the renal capsule and adherent vessels. The kidneys were embedded in 4% low melt agarose (Bio&SELL, BS20.47.100) and then cut into 250 μm thick tissue sections using a Leica VT1200S vibratome (Leica Microsystems), amplitude: 1.0 mm and cutting speed: 0.20 mm/sec. Protected from light, the slices were stored in ice-cold 1X KHB for up to 6 h during the imaging session.

### Custom-built perfusion system

The perfusion system included the microscope stage chamber MS4 (Digitimer) connected through the female luer lock (Drifton, LF31) to the Original Perfusor^®^ tubing line (B. Braun, 8723060). The respective end of the Perfusor^®^ line tubing was inserted into a 1 ml Stripette^®^ (Corning, 4011) to provide a stable support for the inlet and outlet. The system was connected to the Masterflex^®^ silicone hose (Cole-Parmer, 96400-13) and clamped into the pump head with 2 channels (Masterflex^®^ L/S^®^ Easy-Load^®^ II, Cole-Parmer, 77202-50) of the Masterflex^®^ L/S^®^ peristaltic pump (Cole-Parmer, 07555-15). The temperature of the buffer was kept constant at 37 °C with the Truelife Invio BW Double (Mercateo, 615-Y791972) and was monitored with an external temperature probe LOG200 TC 5005 − 0204 (Dostmann Electronic). The Perfusor^®^ line tubing was wrapped with sealing foam tape (SourcingMap) to avoid temperature loss of the heated buffer.

### Real-time imaging of AKS

AKS were transferred onto a poly-L-lysine coated coverslip, positioned centrally in the imaging chamber, and secured with the slice anchor SHD-42/10 (Multi Channel Systems MCS, SKU:641419). Thereafter, the slices were allowed to adapt to the pre-tempered 1X KHB (37 °C) for 10 min at a continuous flow rate of 1 ml/min using the custom-build perfusion system. Stimulants tested on AKS included: 500 nM or 1 µM Atrial natriuretic factor (1-28) trifluoroacetate (ANP, Bachem, 01-4030380); 100 µM Diethylammonium(Z)-1-(N, N-diethylamino)diazen-1-ium-1,2-diolate (DEA NONOate, Biomol, Cay82100-100); 500 µM or 1 mM S-nitroso-N-acetylpenicillamine (SNAP, Biomol, Cay82250). SNAP was chosen for its longer half-life compared to other NO donors, making it well-suited for image acquisition over longer periods of time. However, high concentrations of SNAP (1 mM) are required to elicit a cGMP increase in tissue slices (Supplemental Fig. 2a)^49,50^. SNAP and DEA NONOate stock solutions were diluted in 1X KHB (pH 7.4) and incubated at RT for 10 min and 5 min, respectively, to induce nitric oxide release prior to administration. PDE inhibitors tested included: 250 µM Avanafil (Hölzel Diagnostika, A12619); 50 µM BAY 60-7550 (Biomol, Cay10011135); 250 µM Cilostamide (Hölzel Diagnostika, A13596); 250 µM Roflumilast (Hölzel Diagnostika, A10804); 100 µM PF-04449613 (Sigma-Aldrich, PZ0349); 500 µM 3-Isobutyl-1-methylxanthine (IBMX, Sigma-Aldrich, I5879). Drug exposure and vitality of the tissue was monitored by the delivery and uptake of 1 µM Evans Blue (Sigma-Aldrich, E2129) from the administered solution. Real-time cGMP imaging was performed using the TCS SP8 MP-OPO microscope (Leica Microsystems) and an IR Apo L25x/0.95 W objective in conjunction with the LAS X software (Leica). The objective was equipped with an objective heating collar (OBJ-COLLAR-3342, Okolab) adjusted to 37 °C, which was monitored and controlled by a temperature controller (H401-T-CONTROLLER, Okolab). Biosensor excitation was conducted with a diode laser at 448 nm together with sequential excitation of Evans Blue with the OPSL laser at 552 nm in combination with the TD beam splitter 448/514/552. Due to cGi500 biosensor heterozygosity, real-time imaging of Tie2:Cre/cGi500 and Cdh5:Cre/cGi500 mice was performed with higher laser power compared to homozygous Pod:Cre/cGi500 mice. Simultaneous detection of emission changes was performed at 480 ± 25 nm (CFP), 535 ± 20 nm (YFP) and 685 ± 35 nm (Evans Blue) with a bidirectional scan at 600 Hz and a line average of 4. Time series were recorded with one frame every 10 s for 150 frames, starting with a baseline acquisition of 20 frames.

### FRET imaging data analysis

Sample motion was corrected through the open-source package Galene (realignment mode: warp, points: 10)^51^. The reassembled data was further processed using ImageJ (NIH) by superimposing a region of interest (ROI) over the whole glomerulus. Glomeruli showing morphological abnormalities and positive Evans Blue staining, indicating damage, were excluded from the analysis (Supplemental Fig. 3/4). Baseline normalization (averaged baseline signal: frame 0–20) was applied either to the CFP and YFP emission intensities ($\:\Delta$F/F_o_) or to the CFP/YFP emission ratios ($\:\Delta$R/R_o_) (Supplemental Fig. 5/6). Relative signal changes in $\:\Delta$R/R_o_ (%) reflect changes in the intracellular cGMP concentration. The depicted curves represent the mean of baseline-normalized CFP/YFP emission ratios (FRET ratio) of all analyzed glomeruli. Scatter plots/Scattered bar plots display the maximal $\:\Delta$R/R_o_ response (maximal %signal change relative to baseline) in a selected time period of all analyzed glomeruli. Introduced delay in the cGMP/FRET response (lag time) ensued post substance administration, with dependency on the length of the assembled superfusion system hose (inlet). Lag time correction (stimulation interval + 60 s) was applied uniformly to all displayed time-lapse recordings with specification of the exact stimulation interval (w/o correction) in the figure legend.

### Immunostaining in optically cleared kidney slices

Immunolabeling of cleared kidney slices was based on an adapted protocol by Unnersjö-Jess et al^52^. Freshly prepared AKS from biosensor mice were fixed overnight in 4% PFA (Sigma-Aldrich, P6148) in 1X PBS. The solution was replaced with 1X PBS. Optical clearing was accomplished by immersing the AKS in 1 ml clearing solution (200 mM boric acid (Sigma-Aldrich, B0252), 4% SDS (Sigma-Aldrich, L3771), pH 8.5) at 50 °C for 24 h, followed by a washing step with PBS-T for 5 min (1X PBS with 0.1% Triton X-100 (AppliChem, A4975)). Primary antibodies: GFP-Alexa647 (1:100, rabbit, Thermo Fisher Scientific, A-31852), α-nephrin (1:100, guinea pig, Fitzgerald Industries International, 20R-NP002), CD31/PECAM-1 (1:200, goat, R&D Systems, AF3628). Secondary antibodies: donkey anti-guinea pig Atto594 or donkey anti-goat Atto594 (1:100, coupled in house). Immunolabeling was carried out at 37 °C overnight with a PBS-T washing step in between and at the end of antibody incubation. Afterwards, AKS were embedded in a saturated fructose solution (80.2% D-fructose (Sigma-Aldrich, F0127), 0.5% 1-thioglycerol (Sigma-Aldrich, M1753)) and placed in glass-bottom dishes with a cover slip on top for mounting. Samples were imaged using a Leica TCS SP8 gSTED 3X with a 775 nm STED laser (Leica Microsystems).

### Statistical analysis

Statistical analysis was performed with GraphPad Prism 10.1.0 (GraphPad Software) utilizing a paired, two-tailed Student’s t-test or One-way ANOVA (Tukey´s post hoc test or Dunnett’s T3 post hoc test) to assess differences between groups. Differences between groups were considered statistically significant if *p* < 0.05 (P value style “GP”). The results are reported as the mean ± standard error of the mean (SEM).

## Results

### Differential regulation of cGMP pathways in GECs and podocytes

**Fig. 1:**
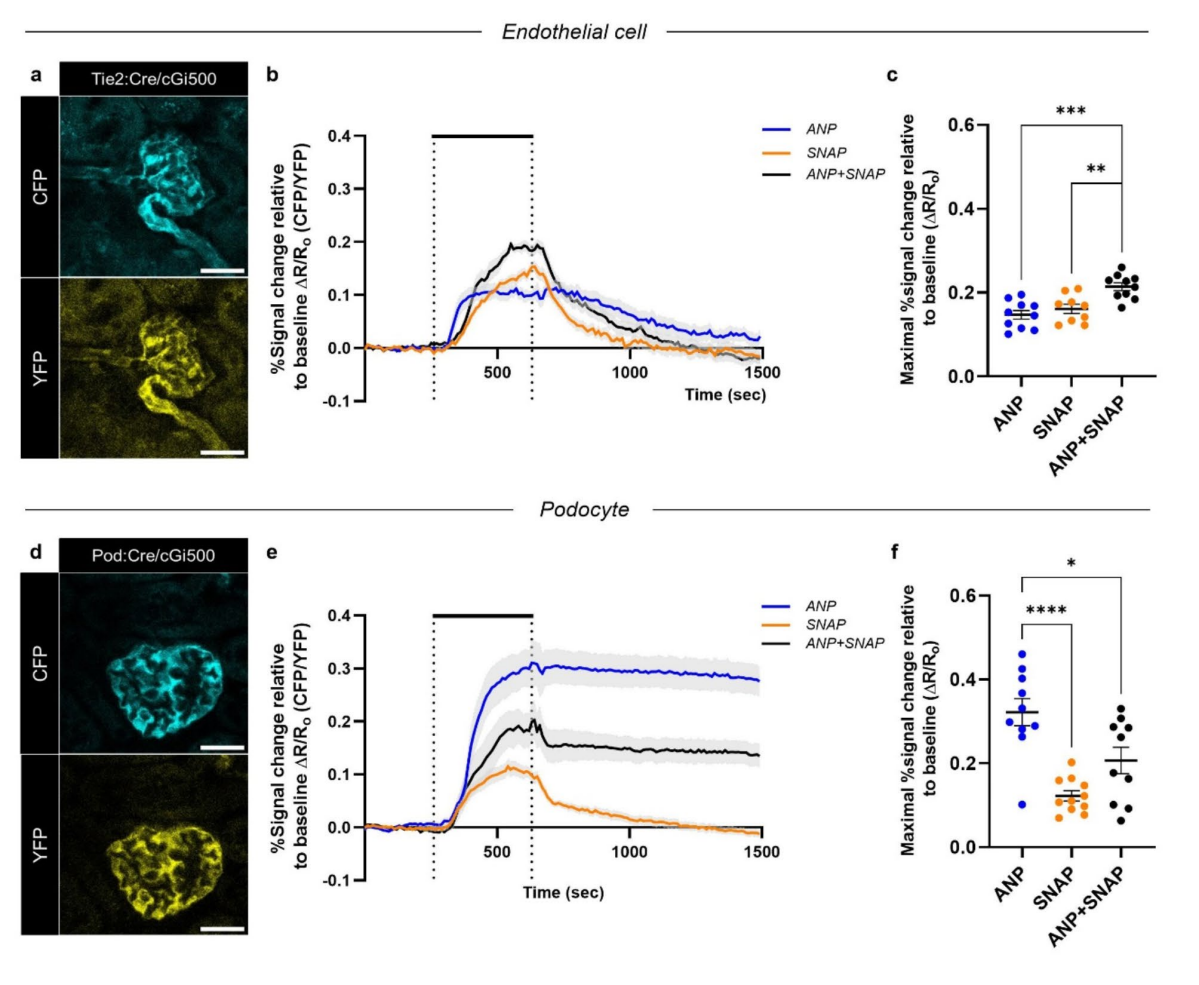
Podocytes respond to ANP/pGC stimulation with a long-lasting cGMP increase. Acute kidney slices obtained from (**a**) Tie2:Cre/cGi500 mice or (**d**) Pod: Cre/cGi500 mice showing cell-specific cGi500 biosensor expression with overlapping CFP/YFP fluorescence patterns in GECs and podocytes. (**b**,** e**) AKS were superfused (dotted lines) from 200 s to 570 s with 1 µM ANP (blue trace), 1 mM SNAP (orange trace), or the combination of both (black trace). Stimulation was followed by a washout phase with 1X KHB (37 °C). Time-lapse recordings for each cell type represent the mean of baseline-normalized FRET (CFP/YFP) ratios ($\:\Delta$R/R_o_) of all analyzed glomeruli. Relative signal changes in ∆R/R_o_ (%) reflect changes in the intracellular cGMP concentration. (**c**,** f**) Scatter dot plots display the maximal ∆R/R_o_ response for each glomerulus derived from the indicated stimulation of (**b**) Tie2:Cre/cGi500 mice or (**e**) Pod:Cre/cGi500 mice during the entire measurement period of 1500 s. Data represent mean ± SEM of at least 9 glomeruli per condition obtained from slices of six Tie2:Cre/cGi500 mice and five Pod:Cre/cGi500 mice. One-way ANOVA, Tukey´s post hoc test, *P < 0.05. Scale bar 25 µM.

Cell-specific cGi500 expression in GECs and podocytes was confirmed by optical tissue clearing and immunofluorescence labeling of AKS (Supplemental Fig. 7). Tie2:Cre/cGi500 and Pod:Cre/cGi500 mice were used to visualize cell-specific changes in the cGMP/FRET response ($\:\varDelta\:$R/R_o_) following the activation of the ANP/pGC/cGMP and NO/sGC/cGMP pathways in glomeruli of freshly prepared kidney slices (Fig. 1). Binding of cGMP to the FRET-based biosensor results in antiparallel intensity changes of the CFP/YFP FRET pair, thereby reporting changes in intracellular cGMP concentration.

In GECs of Tie2:Cre/cGi500 mice, SNAP stimulation (NO Donor, 1 mM) reached the maximal cGMP/FRET response later and declined earlier compared to ANP stimulation (1µM) (Fig. 1b). However, both substances elicited a similar maximal cGMP/FRET response (Fig. 1c). The kinetics of the cGMP response to the co-stimulation with ANP + SNAP was comparable to those observed with SNAP stimulation alone (Fig. 1b). Yet, the combined stimulation had a synergistic effect, significantly elevating the maximal cGMP/FRET response compared to the response induced by each substance alone (Fig. 1c). For Tie2:Cre mouse lines, non-endothelial Cre recombinase activity has been reported^46,47^. To demonstrate endothelial specificity, we repeated the experiments with ANP or/and SNAP in another Cre driver line (Cdh5:Cre/cGi500 mice) and observed a signal time course similar to that of Tie2:Cre/cGi500 mice (Supplemental Fig. 8). To address potential unspecific effects of SNAP attributable to its concentration, we applied another NO donor, DEA NONOate, which triggered a transient signal similar to that of SNAP in GECs of Tie2:Cre/cGi500 and Cdh5:Cre/cGi500 mice (Supplemental Fig. 9)^36,37^.

Podocytes of Pod:Cre/cGi500 mice showed an increase in cGMP levels after the administration of 1 mM SNAP (Fig. 1e, f) with similar kinetics and a maximal cGMP/FRET response compared to GECs (Fig. 1b, c). ANP-triggered cGMP synthesis in podocytes resulted in the highest cGMP/FRET response of all experiments and remained elevated throughout the measurement period (Fig. 1e). This long-lasting plateau receded only after a total recording time of 4250 s ($\:\sim$ 70 min), demonstrated by a long-term measurement (Supplemental Fig. 10), and was also present with half of the ANP concentration (500 nM ANP, Supplemental Fig. 2b). In podocytes, co-stimulation failed to elicit a synergistic additive effect. Instead, the response showed a transient peak, most likely due to NO release from SNAP, followed by a plateau similar to the long-lasting cGMP response observed with ANP alone (Fig. 1e). Notably, the maximal cGMP/FRET response was lower with the combined stimulation compared to ANP alone (Fig. 1f).

Overall, our experiments demonstrate clear differences in the activity of the ANP/pGC/cGMP and NO/sGC/cGMP pathways between GECs and podocytes, and indicate a predominant role of the ANP/pGC/cGMP signaling pathway with sustained cGMP elevation in podocytes.

### cGMP levels are controlled by a higher PDE activity in GECs compared to podocytes

**Fig. 2:**
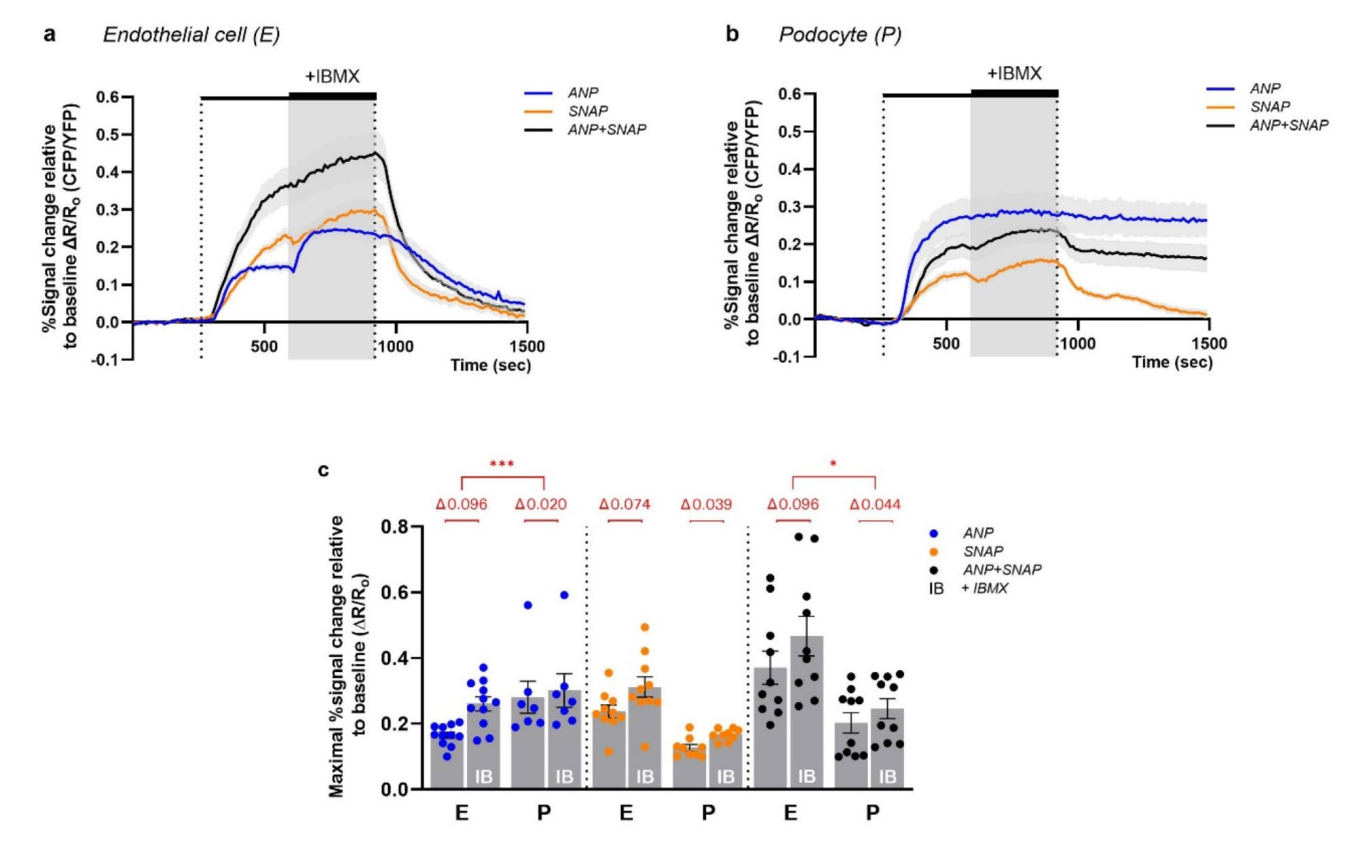
Addition of the PDE inhibitor IBMX reveals higher PDE activity in GECs compared to podocytes. Acute kidney slices from (**a**) Tie2:Cre/cGi500 mice or (**b**) Pod:Cre/cGi500 mice were superfused (outer dotted lines) from 200 s to 860 s with 1 µM ANP (blue trace), 1 mM SNAP (orange trace) or the combination of both (black trace). In addition to the ongoing stimulation, 500 µM IBMX was administered continuously from 530 s to 860 s (gray area). Time-lapse recordings for each cell type represent the mean of baseline-normalized FRET (CFP/YFP) ratios ($\:\Delta$R/R_o_) of all analyzed glomeruli. (**c**) Comparison of the additional cGMP/FRET response after IBMX administration between GECs and podocytes. Scattered bar plot displays the maximal ∆R/R_o_ response for each glomerulus in the respective measurement interval (w/o IBMX: 260–590 s; with IBMX: 600–1500 s). The delta (mean difference, shown in red) between the two time intervals was compared between GECs (E) and podocytes (P). Data represent mean ± SEM of at least 7 glomeruli per condition obtained from slices of three Tie2:Cre/cGi500 mice and four Pod:Cre/cGi500 mice. One-way ANOVA (Brown Forsythe (*p* < 0.0001), Welch test (*p* < 0.0001)), Dunnett T3 post hoc test, *P < 0.05.

The balance of intracellular cGMP concentration within cells or tissues is maintained by the coordinated interplay between cGMP synthesis and its degradation, primarily mediated by phosphodiesterases (PDEs). Therefore, we added the broad-spectrum PDE inhibitor IBMX to the ongoing stimulation with ANP or/and SNAP. In GECs of Tie2:Cre/cGi500 mice, IBMX administration to an ongoing ANP or ANP + SNAP stimulation resulted in a higer additional cGMP/FRET response than the IBMX administration to an ongoing SNAP stimulation (Fig. 2a). The cGMP response elicited by ANP + SNAP (prior to IBMX) stabilizes only during the final seconds of the stimulation (Fig. 2a). In contrast, in podocytes of Pod:Cre/cGi500 mice, the addition of IBMX to an ongoing ANP stimulation led only to a minimal additional increase in the cGMP/FRET response (Fig. 2b), while the addition of IBMX to SNAP or to ANP + SNAP stimulation resulted in a further increase in cGMP levels. However, in all tested combinations, the additional cGMP/FRET response elicited by IBMX was consistently lower in podocytes compared to GECs, which reached statistical significance for ANP and ANP + SNAP (Fig. 2c). These findings suggest that PDE activity is less pronounced in podocytes, while higher constitutive PDE activity in GECs enables a more tight regulation of cGMP levels.

### Predominant PDE2a activity in GECs and PDE3 & PDE5 activity in podocytes

**Fig. 3:**
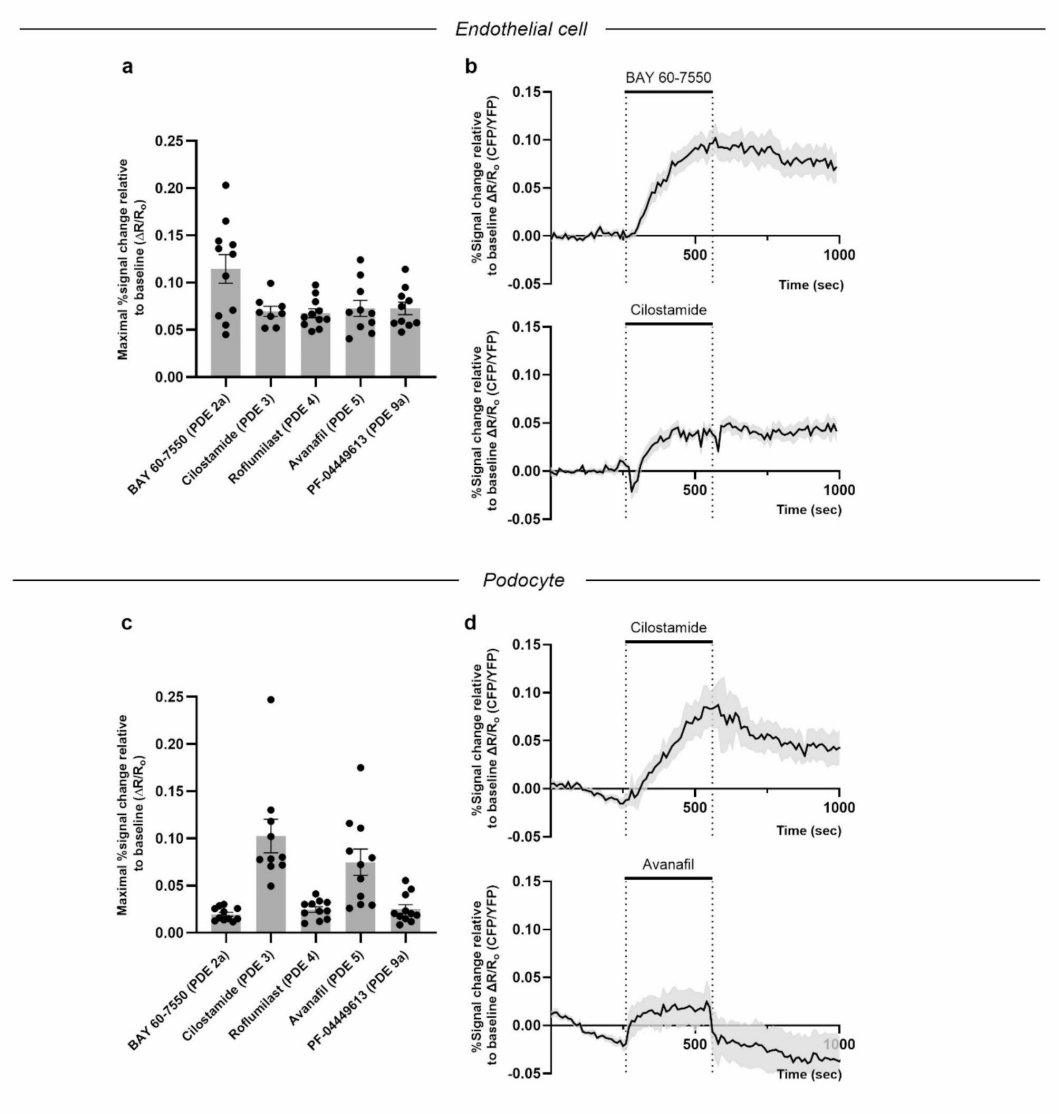
PDE inhibition reveals predominant PDE2a activity in GECs and PDE3 & PDE5 activity in podocytes. Acute kidney slices from (**a**) Tie2:Cre/cGi500 mice or (**c**) Pod:Cre/cGi500 mice were superfused for 370 s with 50 µM BAY 60-7550, 250 µM Cilostamide, 250 µM Roflumilast, 250 µM Avanafil or 100 µM PF-04449613. The primarily inhibited PDE isoform is stated in brackets. Baseline-normalized FRET (CFP/YFP) ratios (R/Ro) of all analyzed glomeruli were calculated. (**b**,** d**) Time-lapse recordings display a sustained cGMP response after BAY 60-7550 and Cilostamide administration in GECs (**b**), whereas Cilostamide and Avanafil lead to a transient cGMP response in podocytes (**d**). Scattered bar plots display the maximal ∆R/Ro response for each compound during the entire measurement period of 1500 s. Data represent mean ± SEM of at least 8 glomeruli per condition obtained from slices of nine Tie2:Cre/cGi500 mice and six Pod:Cre/cGi500 mice.

To further analyze PDE activity in GECs and podocytes, we inhibited several PDE isoforms and investigated their effect on cGMP levels without ANP or/and SNAP stimulation. Suitable PDE candidates were identified by their high expression levels in transcriptomics and proteomics datasets of endothelial cells and podocytes^53–55^. Respective PDE inhibitors were applied on AKS of Tie2:Cre/cGi500 mice (Fig. 3a, b) and Pod:Cre/cGi500 mice (Fig. 3c, d). In GECs, inhibition of PDE2a by BAY 60-7550 alone resulted in the highest maximal cGMP/FRET response of all tested substances. The inhibition of all other PDE isoforms tested (PDE3, 4, 5, 9a) also elicited a robust cGMP/FRET response. In contrast, in podocytes, PDE3 inhibition by Cilostamide induced the highest cGMP/FRET response of all tested substances, followed by PDE5 inhibition with Avanafil. The other PDE inhibitors elicited only a minor cGMP/FRET response. This finding suggests a broader PDE activity in GECs compared to podocytes.

### PDE2a inhibition prevents cGMP degradation in GECs, whereas PDE3 & PDE5 inhibition augment the ANP/pGC/cGMP pathway in podocytes

**Fig. 4:**
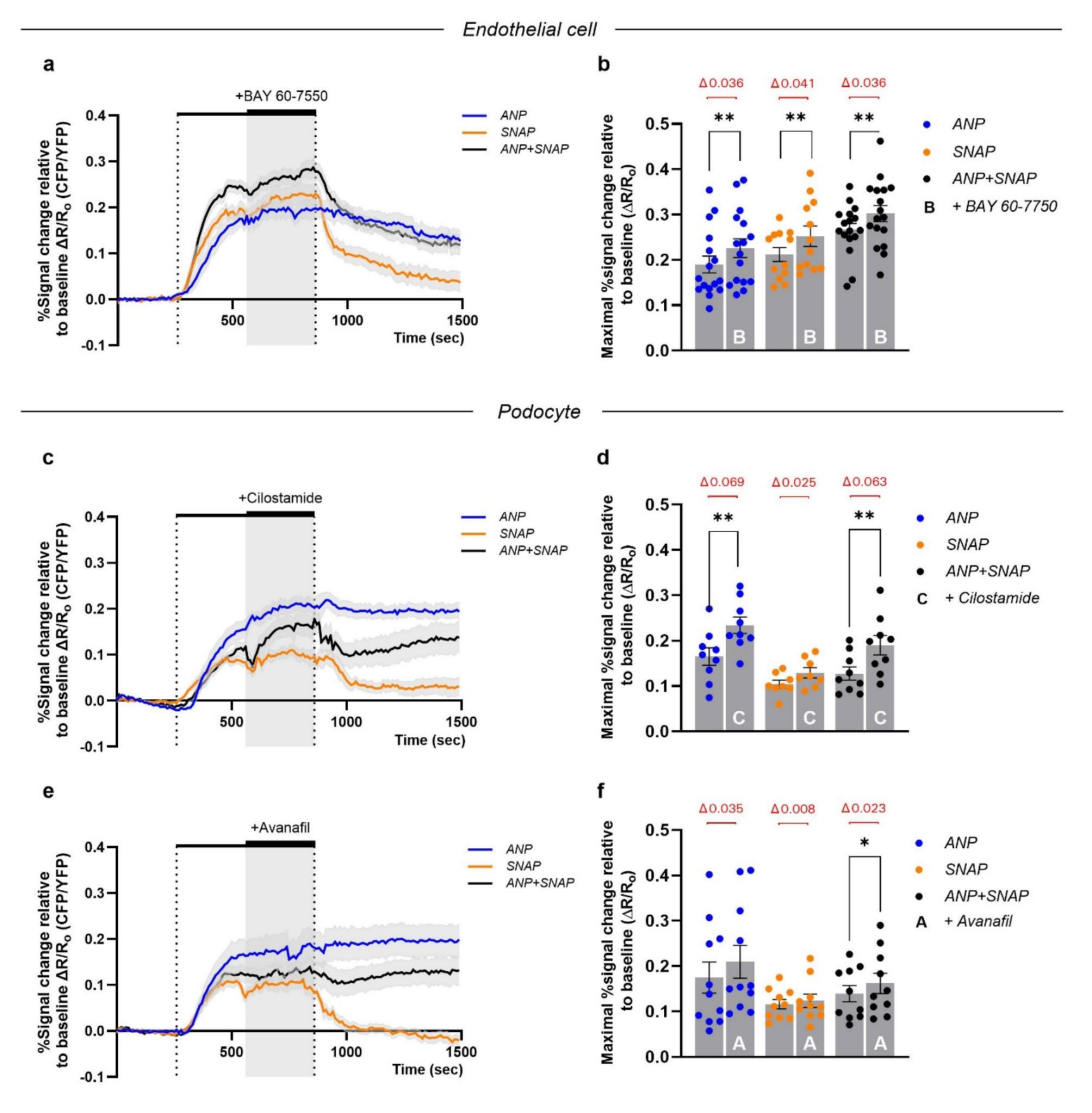
PDE2a inhibition prevents cGMP degradation in GECs, whereas PDE3 & PDE5 inhibition augment the ANP/pGC/cGMP pathway in podocytes. Acute kidney slices from (**a**) Tie2:Cre/cGi500 mice or (**c**,** e**) Pod:Cre/cGi500 mice were superfused from 200 s to 800 s (outer dotted lines) with 1 µM ANP (blue trace), 1 mM SNAP (orange trace) or the combination of both (black trace). In addition to the ongoing stimulation, Tie2:Cre/cGi500 slices were superfused continuously with 50 µM BAY 60-7550 and Pod:Cre/cGi500 slices were superfused with 250 µM Cilostamide or 250 µM Avanafil in the time period from 500 s to 800 s (gray area). Time-lapse recordings represent the mean of baseline-normalized FRET (CFP/YFP) ratios (∆R/R_o_) of all analyzed glomeruli. (**b**,** d**,** f**) Scattered bar plots display the maximal ∆R/R_o_ response for each glomerulus in the respective measurement interval (w/o PDE inhibitor: 200–570 s; with PDE inhibitor: 580–950 s) for (**b**) BAY 60-7550, (**d**) Cilostamide or (**f**) Avanafil. The delta (mean difference) between the two time periods is shown in red. Data represent mean ± SEM of at least 8 glomeruli per condition obtained from slices of eight Tie2:Cre/cGi500 mice after administration of BAY 60-7550, eight Pod:Cre/cGi500 mice for Cilostamide and nine Pod:Cre/cGi500 mice for Avanafil. Two-tailed paired t-test. *P < 0.05.

To investigate the effect of PDE inhibition on the ANP/pGC/cGMP and NO/sGC/cGMP pathways, we applied inhibitors of specific PDE isoforms to an ongoing stimulation with ANP or/and SNAP on AKS of Tie2:Cre/cGi500 mice or Pod:Cre/cGi500 mice. In GECs of Tie2:Cre/cGi500 mice, the administration of BAY 60-7550 (PDE2a inhibitor) resulted in an additional cGMP/FRET response during ANP or/and SNAP stimulation (Fig. 4a) with a similar effect strength (Fig. 4b). However, the increased cGMP levels lasted considerably longer than the cGMP/FRET response after ANP or/and SNAP stimulation without PDE inhibition, as none of the FRET ratios returned back to baseline within the measurement period (Fig. 4a). To study the effect of inhibiting specific PDE isoforms in podocytes, we applied Cilostamide (PDE3 inhibitor) or Avanafil (PDE5 inhibitor) on AKS of Pod:Cre/cGi500 mice. Both substances showed a pronounced effect on the ANP and ANP + SNAP stimulation (Fig. 4c-f). However, the additional cGMP/FRET response after SNAP stimulation was small for Cilostamide (Fig. 4d) and almost absent for Avanafil (Fig. 4f). The plateau-like cGMP response seen after ANP and ANP + SNAP stimulation was unaffected by inhibition of PDE3 or PDE5.

Taken together, we were able to demonstrate clear pathway-dependent differences in the activity of PDE isoforms between GECs and podocytes.

## Discussion

To our knowledge, our study is the first, which resolves a cell-specific cGMP response in glomerular cells in vital kidney tissue. For podocytes, we demonstrate an elevation of intracellular cGMP levels provoked by the stimulation of the ANP/pGC/cGMP-axis and NO/sGC/cGMP-axis. These findings contradict the work of Theilig et al. and Wang et al., who were unable to detect SNAP-induced cGMP synthesis in cultured podocytes^31,32^, but are in line with results from other authors^33,34,56,57^. However, limitations in conventional detection and quantification methods in these publications can hamper the assessment of cGMP dynamics^38^. Additionally, the severe differences in gene expression and morphology between cultured podocytes and podocytes in vivo must be taken into account^58,59^. In agreement with our results, Mundel et al. and Jarry et al. localized sGC in podocytes of kidney tissue^42,60^.

Endothelial cells are known NO suppliers, but there is limited data on the endogenous expression of guanylyl cyclases (sGC or pGC) and cGMP signaling in GECs of intact tissue^34,40–42,61^. Our data provide evidence for the existence of the ANP/pGC/cGMP and NO/sGC/cGMP pathways in GECs. We demonstrate that GECs possess an actively regulated cGMP signaling system, measured with high spatial and temporal resolution in glomeruli embedded in a complex kidney architecture. Compared to conventional assays that rely on single cGMP endpoint determination, our experimental setup enabled real-time monitoring of cGMP dynamics throughout the study period.

The FRET-based cGMP biosensor utilized here was capable of detecting small changes in intracellular cGMP concentrations (EC50$\:\:\sim\:$532 nM, Supplemental Fig. 1), and its functionality has been validated in other ex vivo settings^36,39,62^. However, during in vivo imaging of the kidney (data not shown), the small FRET changes of the sensor, further attenuated by the expression in only a subset of glomerular cells, were concealed by artifacts of respiratory movements of the animals. Therefore, we could only conduct experiments ex vivo on AKS. But as a trade-off, we had to apply higher doses than those published for cell culture to achieve the required penetration, a practice commonly adopted for tissue slices^50^. Based on our experience, in vivo imaging of the kidney with the presently available cGMP biosensor mouse lines is not feasible. The low signal-to-noise ratio associated with the widely used CFP/YFP FRET pair necessitates the use of high-intensity excitation, which carries a risk of phototoxicity and potential damage to kidney tissue^63^. Further improvement of the ratiometric FRET sensor could facilitate cGMP measurements during in vivo imaging.

While previous studies have utilized the ubiquitous expression of the cGMP biosensor in the kidney^39^, our cell-specific approach demonstrates distinct differences in cGMP signaling between GECs and podocytes. In podocytes, SNAP-induced cGMP synthesis resulted in a transient cGMP/FRET response, similar to ANP or/and SNAP stimulation in GECs. In contrast, ANP-induced cGMP synthesis in podocytes resulted in a higher and far longer lasting cGMP increase than in GECs. The persistent ANP-triggered cGMP response in podocytes did not decline to baseline level during a total measurement time of 4250 s ($\:\sim$70 min) (Supplemental Fig. 10). Reducing the ANP concentration by 50% resulted in an identically sustained cGMP/FRET response, albeit at a lower level (Supplemental Fig. 2b), which argues against plateau formation due to oversaturation of the sensor. This observation could be explained by the delayed internalization of ligand-receptor complexes of GC-A^64,65^, as podocytes exhibit high expression levels of GC-A^41^. Alternatively, this phenomenon may arise from a positive feedback loop via PKG^16^, an inhibitory effect of cGMP concentrations on PDE3 activity^66,67^, or generally low PDE activity, as demonstrated in our experiments. These results suggest an important role for ANP-mediated cGMP synthesis in podocytes, which has been demonstrated in disease states for a podocyte-specific GC-A knockout model. In these mice, DOCA salt-induced hypertension led to the development of proteinuria and foot process effacement^68^. In all of our experiments, the co-stimulation (ANP+SNAP) in podocytes resulted in a lower cGMP increase than the stimulation with ANP alone, whereas it had an additive effect in GECs. Similar results for podocytes regarding a possible mutual regulation of sGC and pGC activity have been published and suggest the avoidance of excessive cGMP concentrations in podocytes^69,70^.

The inhibition of PDE isoforms revealed substantial differences in their activity between GECs and podocytes. In GECs, all tested PDE inhibitors (targeting PDE2a, PDE3, PDE4, PDE5 and PDE9a) increased basal cGMP levels, while PDE2a inhibition had the strongest effect. In podocytes, only the PDE3 inhibitor Cilostamide and the PDE5 inhibitor Avanafil resulted in comparable increases in basal cGMP levels. Remarkably, all tested PDE inhibitors, including the non-specific PDE inhibitor IBMX (Supplemental Fig. 11), induced a sustained cGMP response in endothelial cells, whereas podocytes exhibited a transient cGMP increase. Inhibition of PDE2a in endothelial cells, following ANP or/and SNAP stimulation, delayed cGMP degradation, while inhibition of PDE 3 and PDE5 in podocytes predominantly augmented ANP-induced cGMP synthesis without affecting the long-lasting cGMP/FRET response in the washout-phase. As a limitation, the applied PDE inhibitors predominantly inhibit one PDE isoform but are not completely specific. Hence, it cannot be excluded that these inhibitors partially influence other PDE isoforms. Administration of the unspecific PDE inhibitor IBMX to podocytes resulted in only a small additional increase in cGMP levels, with a greater effect on the NO/sGC/cGMP-pathway. The less pronounced effect of unspecific PDE isoform inhibition by IBMX on cGMP levels compared to that of specific PDE isoform inhibition might be explained by the unequally effective inhibition of different PDE isoforms by IBMX^71,72^. Taken together, these findings demonstrate that GECs and podocytes rely on distinct PDE isoforms and that their impact varies between the two cGMP pathways, which mandates studying glomerular cGMP signaling with high spatial and temporal resolution. Future investigations focusing on additional glomerular cell types, such as mesangial cells, which also possess the capacity to synthesize cGMP^73,74^, are necessary to provide a comprehensive understanding of renal cGMP physiology.

Regarding the limitations of this study, our experiments were not designed to investigate downstream targets of the cGMP signaling pathway, which could explain the nonadditive effect of ANP + SNAP stimulation or the long-lasting effect of ANP stimulation in podocytes. Due to the direct application of ANP or/and SNAP, our study cannot reveal whether the physiologic stimulation of the ANP/pGC/cGMP and NO/sGC/cGMP pathways in GECs and podocytes occurs through autocrine or paracrine signaling mechanisms. The observed data variability could arise from both reagent accessibility to individual glomeruli and chemical batch effects. We therefore ensured that the same batch of each reagent was used for all experiments on podocytes and GECs within each figure.

Overall, we demonstrated the presence of the ANP/pGC/cGMP pathway as well as the NO/sGC/cGMP pathway in GECs and podocytes of healthy glomeruli. ANP stimulation resulted in higher and longer-lasting cGMP production in podocytes compared to SNAP stimulation and the cGMP response triggered by ANP and/or SNAP in GECs. PDE activity in podocytes is dominated by PDE3 and PDE5, while in GECs, all tested PDE isoforms are active, and inhibition of PDE2a had the strongest effect.

Due to the existing controversies in the literature, we intentionally focused on cGMP signaling in healthy conditions. As cGMP signaling is downregulated in disease states and pharmacological manipulation intends to restore cGMP pathways, further studies with different disease models are warranted to increase our understanding of disease-associated changes in cGMP signaling in glomerular cells.

## Electronic supplementary material

Below is the link to the electronic supplementary material.

Supplementary Material 1

## Data Availability

The authors confirm that the data supporting the findings of this study are available within the article and/or its supplementary materials. The datasets generated during and/or analyzed during the current study are available from the corresponding author on reasonable request.
