# Supplementary material for: Real-time imaging of cGMP signaling shows pronounced differences between glomerular endothelial cells and podocytes
Source: Sci Rep. 2024 Oct 30;14:26099. doi: 10.1038/s41598-024-76768-1 (PMC11525973; doi:10.1038/s41598-024-76768-1)
Supplement: Supplementary file 1 — Supplementary Material 1 [file 41598_2024_76768_MOESM1_ESM.pdf]

Real-time imaging of cGMP signaling shows pronounced differences between glomerular endothelial cells and podocytes

Supporting information

Content: 11 Supplemental figures

Supplemental Figure 1: *In vitro* characterization of cGi500.

Supplemental Figure 2: High doses of ANP and SNAP are required to elicit measurable cGi500-mediated cGMP signals.

Supplemental Figure 3: Visualization of the cutting process-related damage to glomeruli.

Supplemental Figure 4: Evans Blue interferes at high concentrations with the FRET-based biosensor cGi500.

Supplemental Figure 5: Calculated FRET (CFP/YFP) ratio from CFP and YFP fluorescence traces demonstrates cGMP generation after activation of the ANP/pGC or/and NO/sGC pathway in GECs.

Supplemental Figure 6: Calculated FRET (CFP/YFP) ratio from CFP and YFP fluorescence traces demonstrates cGMP generation after activation of the ANP/pGC or/and NO/sGC pathway in podocytes.

Supplemental Figure 7: Cell-specific expression of the cGMP biosensor in optically cleared AKS.

Supplemental Figure 8: GECs of Cdh5:Cre/cGi500 mice exhibit a transient response upon stimulation of the ANP/pGC/cGMP and NO/sGC/cGMP pathway.

Supplemental Figure 9: DEA NONOate evokes similar cGMP responses in GECs of Cdh5:Cre/cGi500 and Tie2:Cre/cGi500 mice.

Supplemental Figure 10: Long-term measurement of the cGMP/FRET response in podocytes after ANP administration.

Supplemental Figure 11: Impact of unspecific PDE Inhibition on cGMP signaling dynamics in endothelial cells and podocytes.

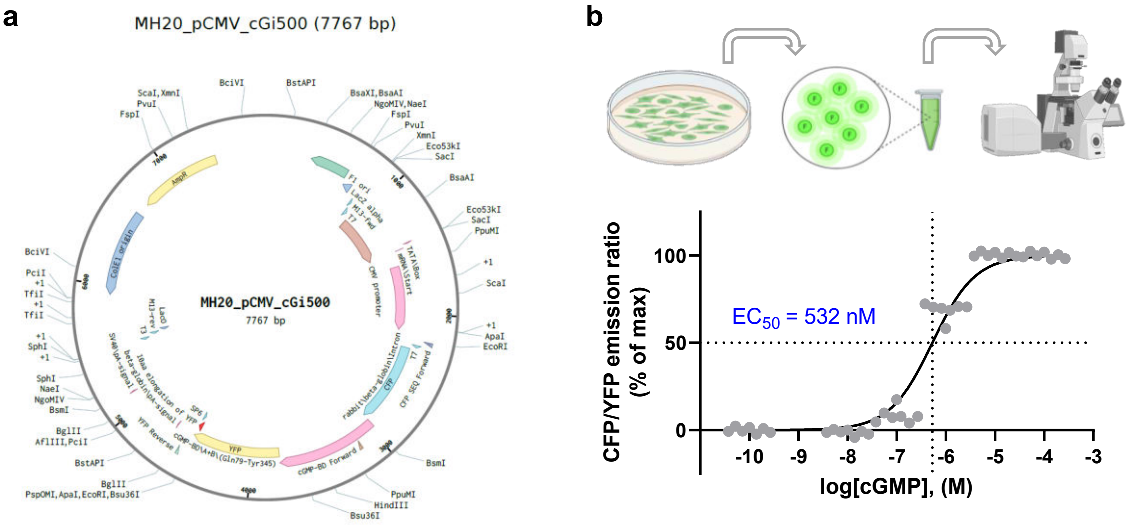

Supplemental Figure 1: *In vitro* characterization of cGi500. (**a**) Plasmid map of MH20_pCMV_cGi500 encoding the monomolecular FRET-based cGMP biosensor cGi500. (**b**) Concentration-response curve analysis of cGi500-transfected HEK 293T cell homogenates. Fluorescence measurements started with the highest cGMP concentration (100 µM cGMP) and progressed to the lowest. Biosensor excitation was conducted with a diode laser at 405 nm together with a dichroic beam splitter (MBS-405) and simultaneous detection of CFP and YFP emission at 480 ± 25 nm and 535 ± 20 nm. The FRET (CFP/YFP) ratio changes were normalized to the first value (absence of cGMP) and the largest value (highest cGMP concentration) in the data set (Hill Slope = 1.0). Data are displayed for each replicate of seven independent experiments.

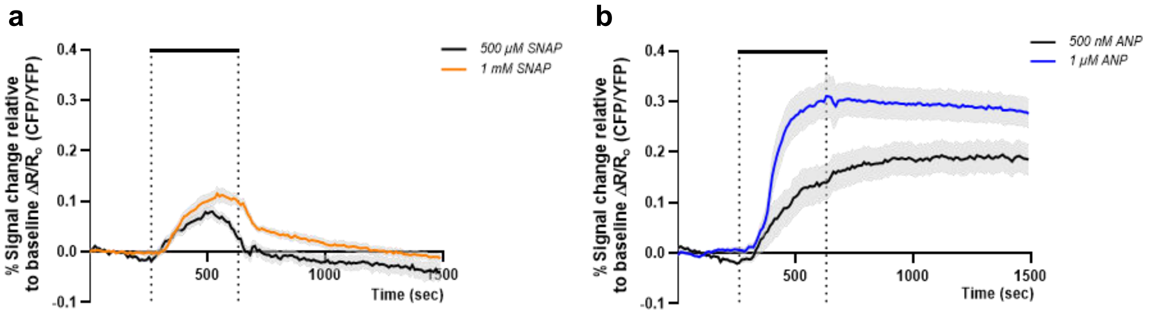

Supplemental Figure 2: **High doses of ANP and SNAP are required to elicit measurable cGi500-mediated cGMP signals**. Acute kidney slices from Pod:Cre/cGi500 mice were superfused (dotted lines) in the time period from 200 sec to 570 sec with (**a**) 1 mM SNAP (orange trace) and 500 µM SNAP (black trace) or (**b**) 1 µM ANP (blue trace) and 500 nM ANP (black trace). Stimulation was followed by pre-tempered 1X KHB (37°C), a time frame of 1500 sec was recorded. Graphs represent the mean of baseline-normalized FRET (CFP/YFP) ratios ($\Delta R/R_o$) of all analyzed glomeruli. Data represent mean ± SEM of at least 10 glomeruli per condition obtained from slices of eight Pod:Cre/cGi500 mice.

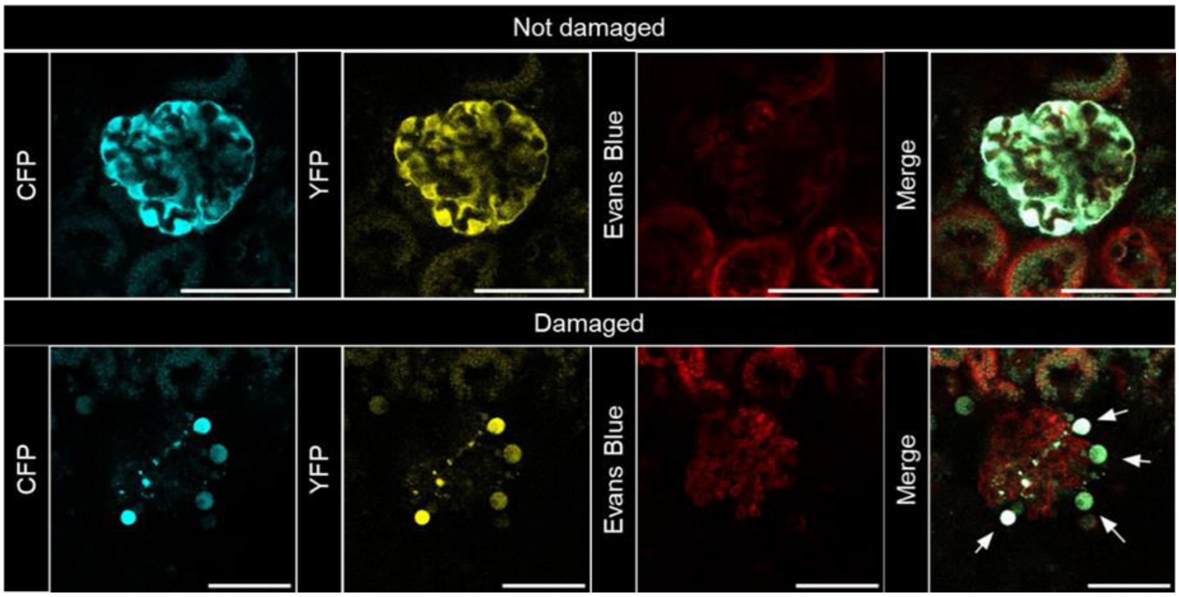

Supplemental Figure 3: Visualization of the cutting process-related damage to glomeruli. (**Top row**) Undamaged glomerulus shown in the CFP (cyan), YFP (yellow), Evans Blue (red) channel and the overlay of all channels (merge). The fluorescence in the Evans blue channel is due to expression of mTomato in all Cre-negative cells. (**Bottom row**) A glomerulus severely damaged by the cutting process with markedly deformed cells (white arrows). The uptake of Evans Blue into the glomerulus is visible in the red channel. Scale bar 50 µM.

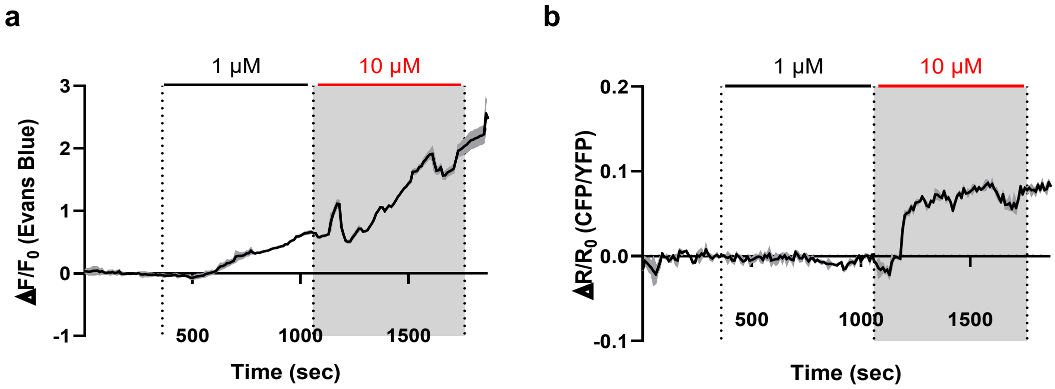

Supplemental Figure 4: Evans Blue interferes at high concentrations with the FRET-based biosensor cGi500. Acute kidney slice of a Pod:Cre/cGi500 mouse superfused with 1 µM and 10 µM Evans Blue solution (dotted lines) while recording time-lapse images. (**a**) While the fluorescence ($\Delta F/F_o$) in the Evans Blue channel increases after superfusion with 1 µM Evans Blue, a non-specific increase in the (**b**) FRET (CFP/YFP) ratio ($\Delta R/R_o$) is only detectable after exposure to 10 µM Evans Blue. All experiments shown in this manuscript were conducted with 1 µM Evans Blue. Data represent mean ± SEM of three glomeruli.

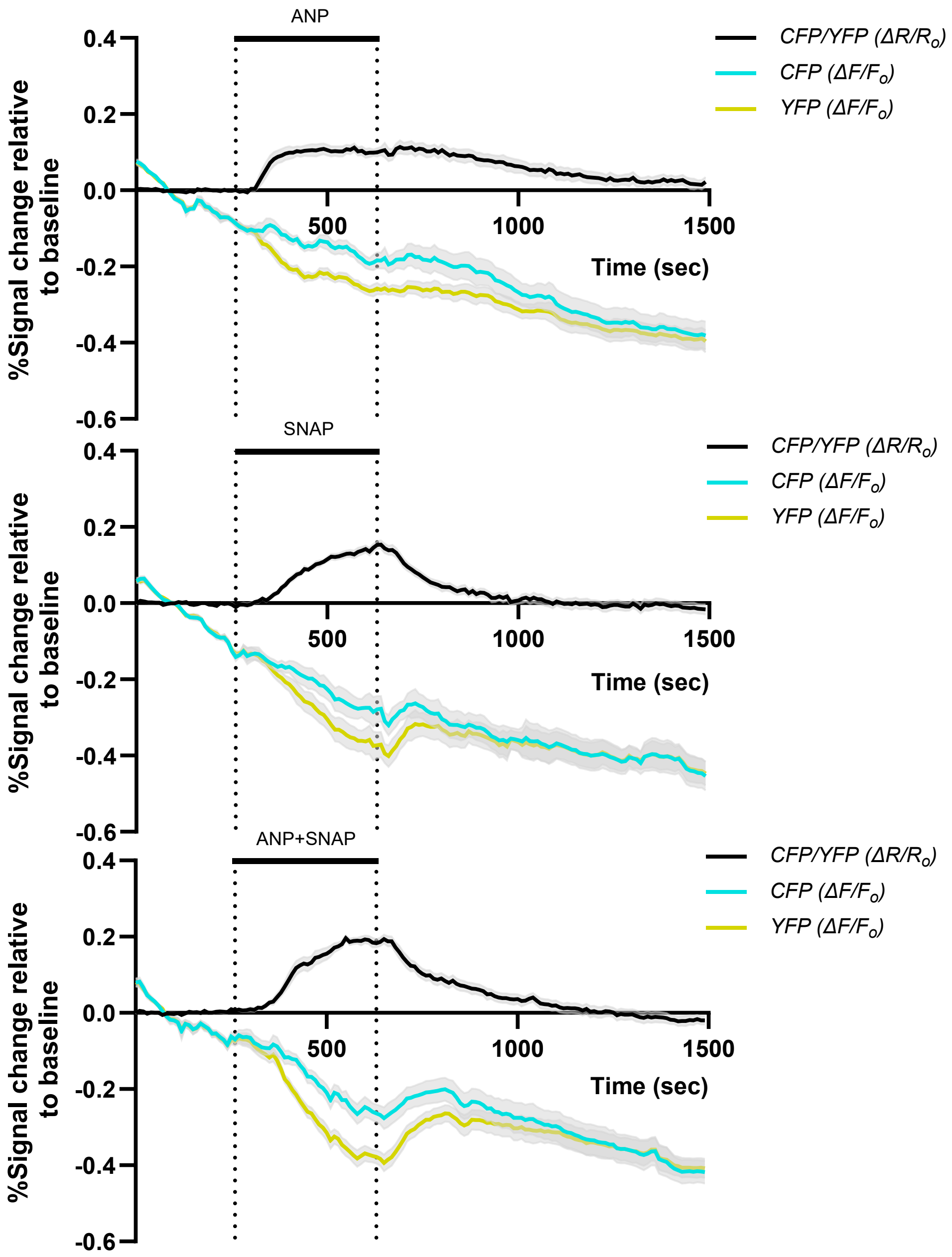

Supplemental Figure 5: Calculated FRET (CFP/YFP) ratio from CFP and YFP fluorescence traces demonstrates cGMP generation after activation of the ANP/pGC or/and NO/sGC pathway in GECs. Acute kidney slices from Tie2:Cre/cGi500 mice were subjected to stimulation with either 1 µM ANP, 1 mM SNAP or a combination of both in the time period from 200 sec to 570 sec (dotted line). Displayed is the emission intensity change over a measurement period of 1500 sec with visualization of the individual CFP (cyan) and YFP (yellow) traces ($\Delta F/F_o$) and the FRET (CFP/YFP) ratio (black, $\Delta R/R_o$). Data represent mean ± SEM of at least 9 glomeruli per condition obtained from slices of six Tie2:Cre/cGi500 mice.

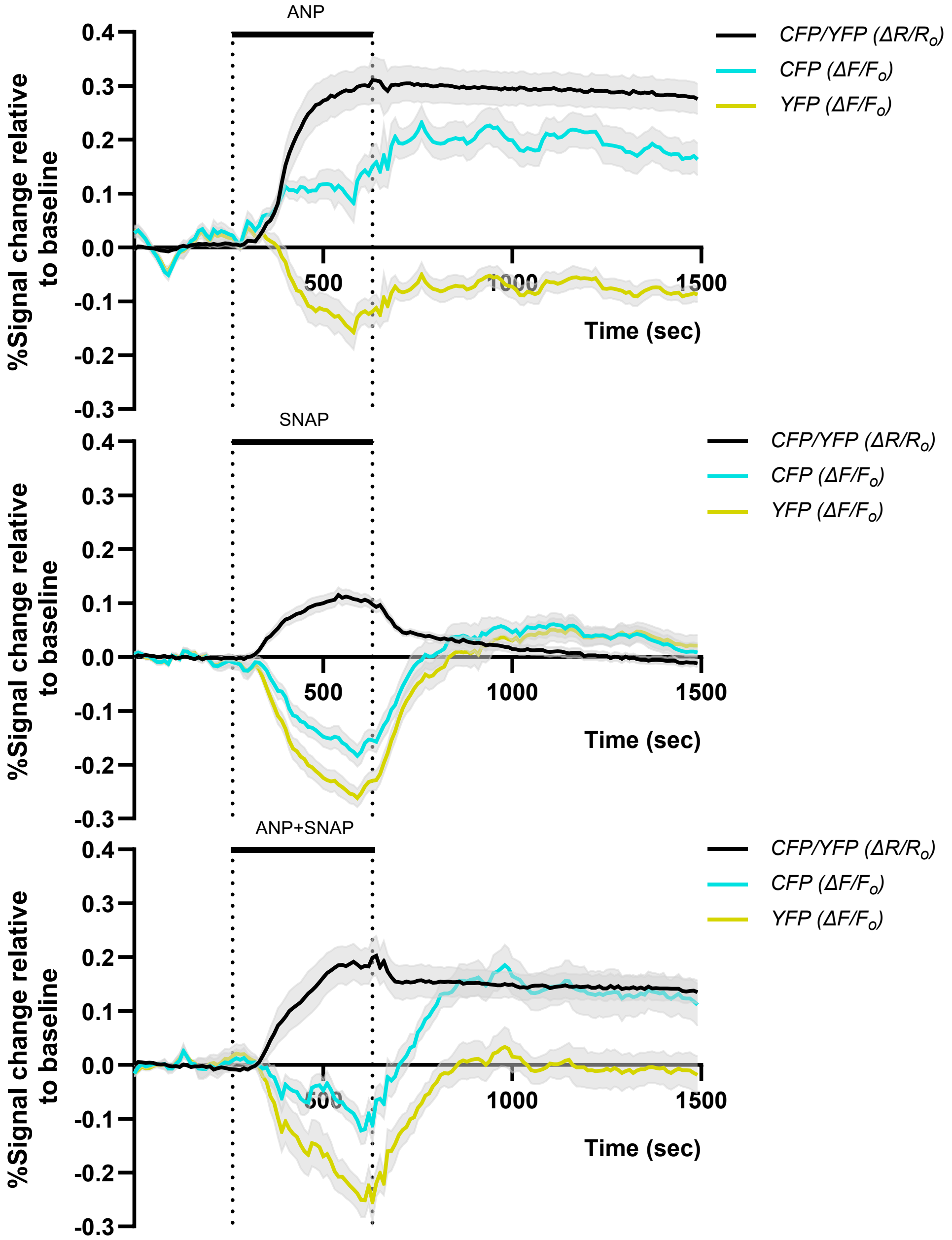

Supplemental Figure 6: Calculated FRET (CFP/YFP) ratio from CFP and YFP fluorescence traces demonstrates cGMP generation after activation of the ANP/pGC or/and NO/sGC pathway in podocytes. Acute kidney slices from Pod:Cre/cGi500 mice were subjected to stimulation with either 1 µM ANP, 1 mM SNAP or a combination of both in the time period from 200 sec to 570 sec (dotted line). Displayed is the emission intensity change over a measurement period of 1500 sec with visualization of the individual CFP (cyan) and YFP (yellow) traces ($\Delta F/F_o$) and the FRET (CFP/YFP) ratio (black, $\Delta R/R_o$). Data represent mean ± SEM of at least 10 glomeruli per condition obtained from slices of five Pod:Cre/cGi500 mice.

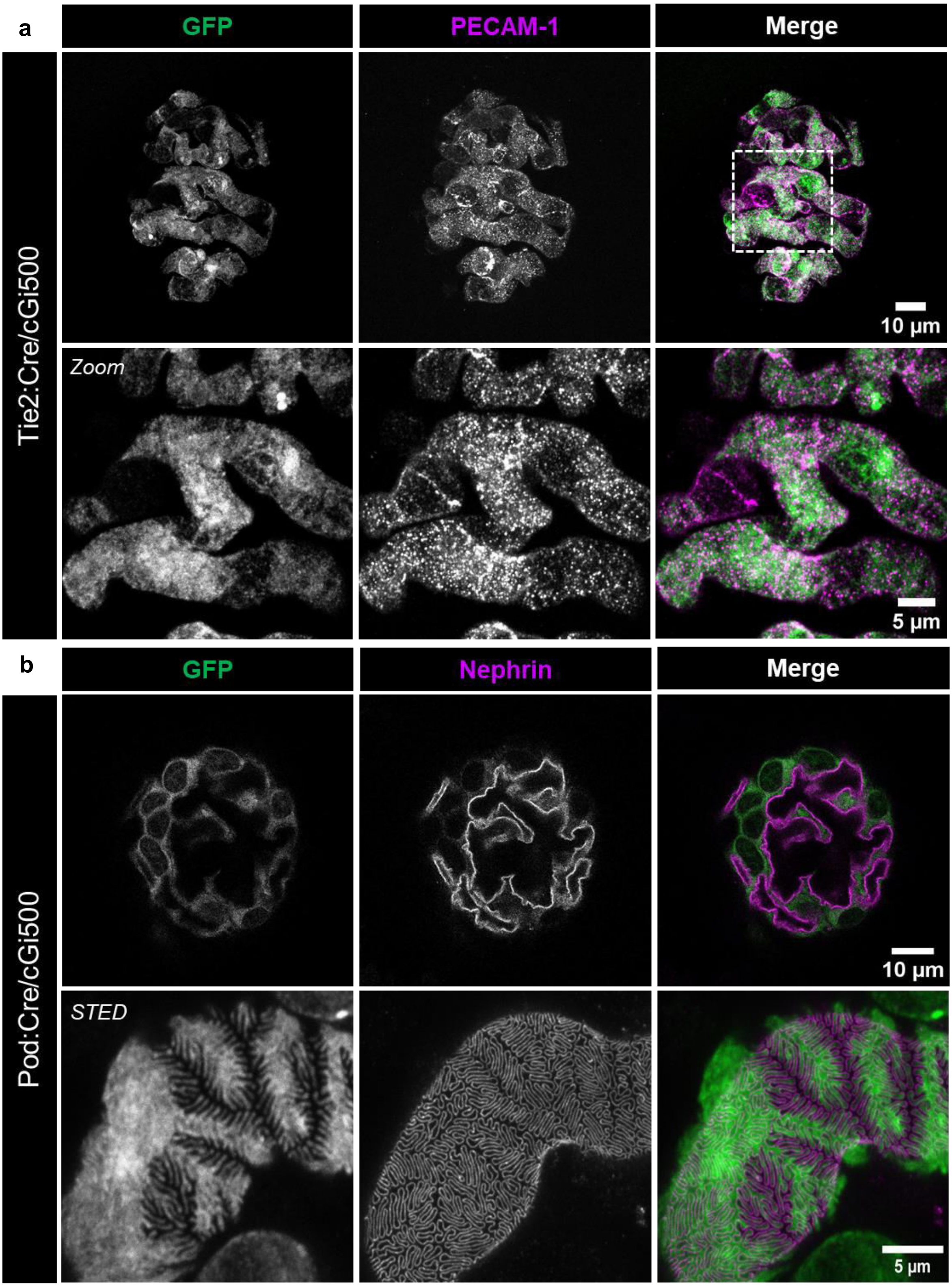

Supplemental Figure 7: Cell-specific expression of the cGMP biosensor in optically cleared AKS. (**a**) Tie2:Cre/cGi500: Confocal images show GFP staining (green) in GECs, thereby confirming cGi500 expression in this cell type. PECAM-1 staining (magenta) functions as a cell-specific marker for GECs and colocalizes with GFP-stained GECs. Bottom panels: High magnification of the area marked with a dashed rectangle in the

right upper panel (Zoom). Both panels display a maximum intensity projection of a Z-stack. (**b**) Pod:Cre/cGi500: Confocal images demonstrate positive GFP staining (green) of podocytes, confirming the presence of biosensor cGi500 expression and overlapping nephrin staining (magenta), which labels the slit diaphragm. Higher magnification STED images (maximum intensity projection of Z-stacks) show a capillary area surrounded by podocytes (green) with characteristic slit diaphragm morphology (magenta).

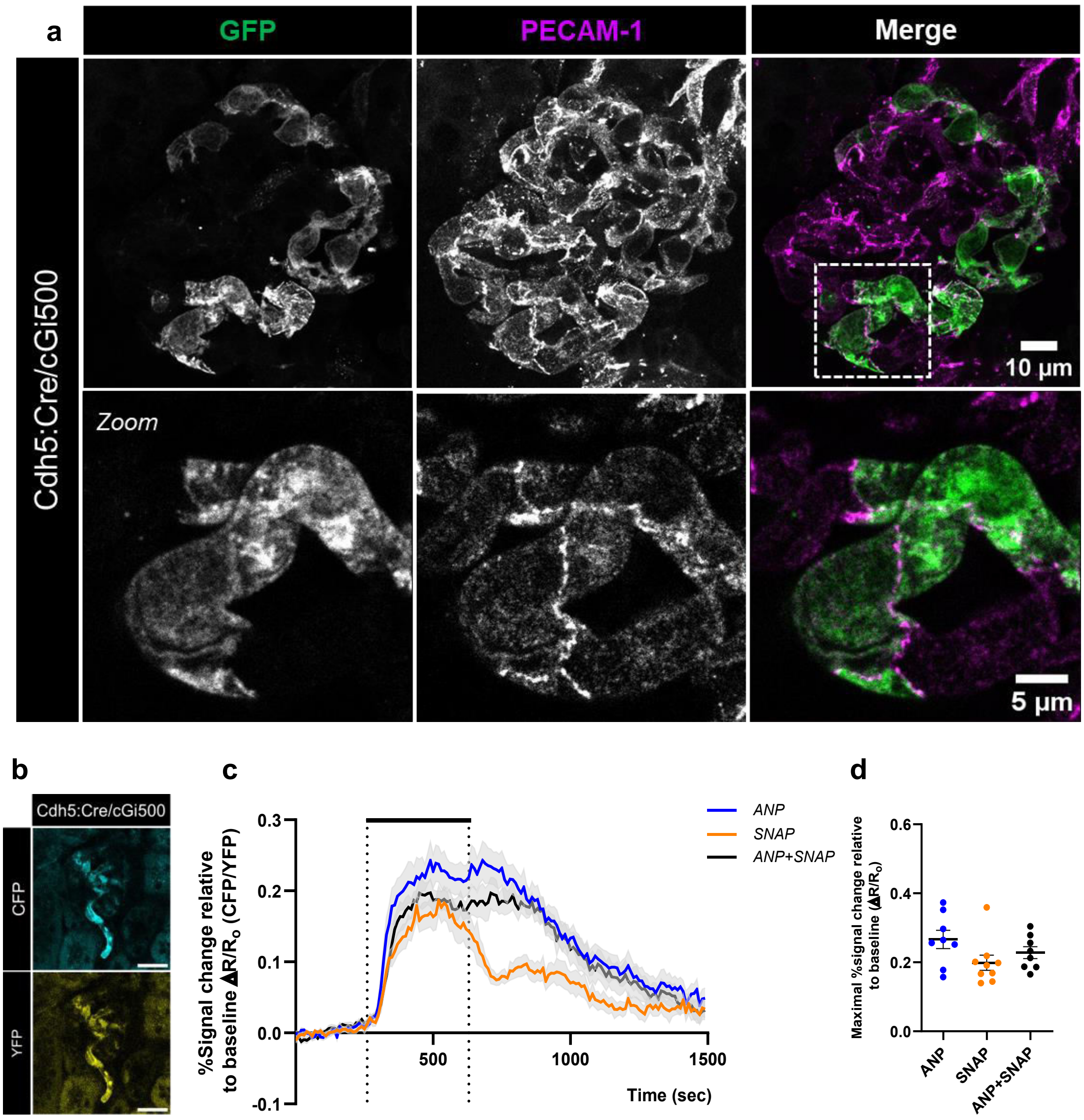

Supplemental Figure 8: GECs of Cdh5:Cre/cGi500 mice exhibit a transient response upon stimulation of the ANP/pGC/cGMP and NO/sGC/cGMP pathway. (**a**) Immunostained kidney slice of a Cdh5:Cre/cGi500 mouse. Confocal images show GFP staining (green) in GECs, thereby confirming cGi500 expression in this cell type. PECAM-1 staining (magenta) functions as a cell-specific marker for GECs and colocalizes with GFP-stained GECs. Bottom panels: High magnification of the area marked with a dashed rectangle in the right

upper panel (Zoom). (**b, c**) Acute kidney slices of Cdh5:Cre/cGi500 mice were superfused (dotted lines) in the time period from 200 sec to 570 sec with 1 µM ANP (blue trace), 1 mM SNAP (orange trace), or the combination of both (black trace). Stimulation was followed by pre-tempered 1X KHB (37°C), a time frame of 1500 sec was recorded. Graphs represent the mean of baseline-normalized FRET (CFP/YFP) ratios ($\Delta R/R_o$) of all analyzed glomeruli. (**d**) Scatter dot plot displays the maximal $\Delta R/R_o$ response for each glomerulus derived from the indicated stimulation in (**c**) during the entire measurement period of 1500 sec. Data represent mean ± SEM of at least 8 glomeruli per condition obtained from slices of five Cdh5:Cre/cGi500 mice. One-way ANOVA, Tukey´s post hoc test, *P < 0.05. Scale bar 25 µM.

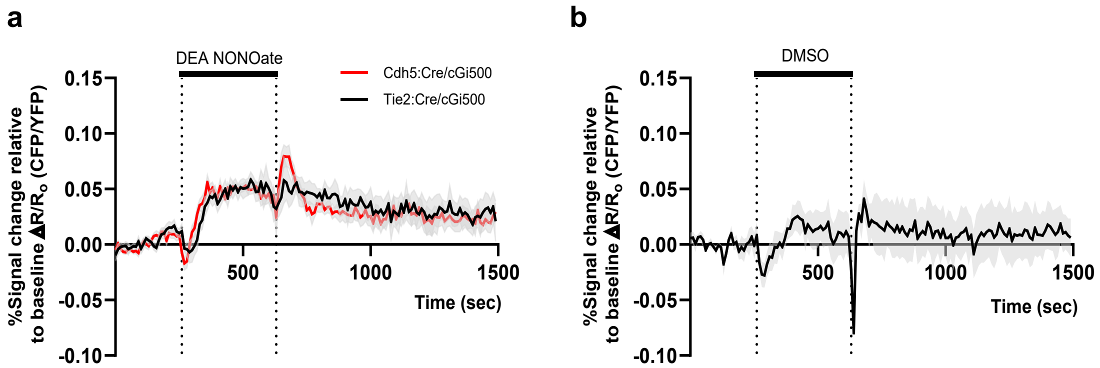

Supplemental Figure 9: DEA NONOate evokes similar cGMP responses in GECs of Cdh5:Cre/cGi500 and Tie2:Cre/cGi500 mice. Acute kidney slices from Cdh5:Cre/cGi500 mice (red trace) or Tie2:Cre/cGi500 mice (black trace) were superfused (dotted lines) with (**a**) 100 µM DEA NONOate or (**b**) DMSO (vehicle control, Tie2:Cre/cGi500) in the time period from 200 sec to 570 sec. Stimulation was followed by pre-tempered 1X KHB (37°C), a total time frame of 1500 sec was recorded. Graphs represent the mean of baseline-normalized FRET (CFP/YFP) ratios ($\Delta R/R_o$) of all analyzed glomeruli. Data represent mean ± SEM of at least 7 glomeruli (DEA NONOate) and 4 glomeruli (DMSO Ctr.) obtained from slices of four Cdh5:Cre/cGi500 mice and two Tie2:Cre/cGi500 mice.

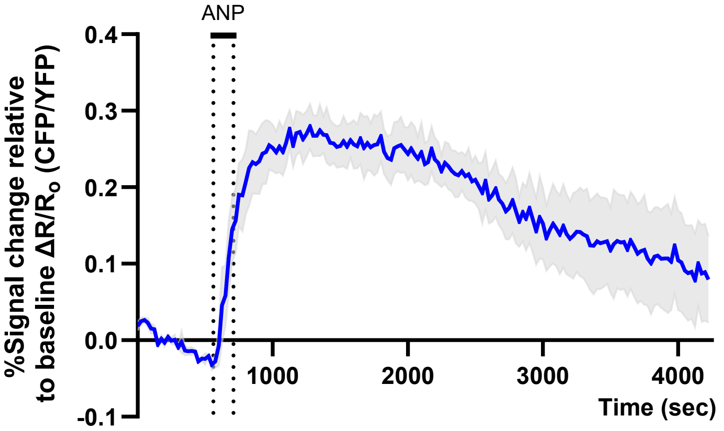

Supplemental Figure 10: Long-term measurement of the cGMP/FRET response in podocytes after ANP administration. Acute kidney slices from Pod:Cre/cGi500 mice were subjected to stimulation with 1 µM ANP in the time period from 500-650 sec. Stimulation was followed by pre-tempered 1X KHB (37°C) for a total recording time of 4250 sec. The graph represents the mean of baseline-normalized FRET (CFP/YFP) ratios ($\Delta R/R_o$) of 4 analyzed glomeruli.

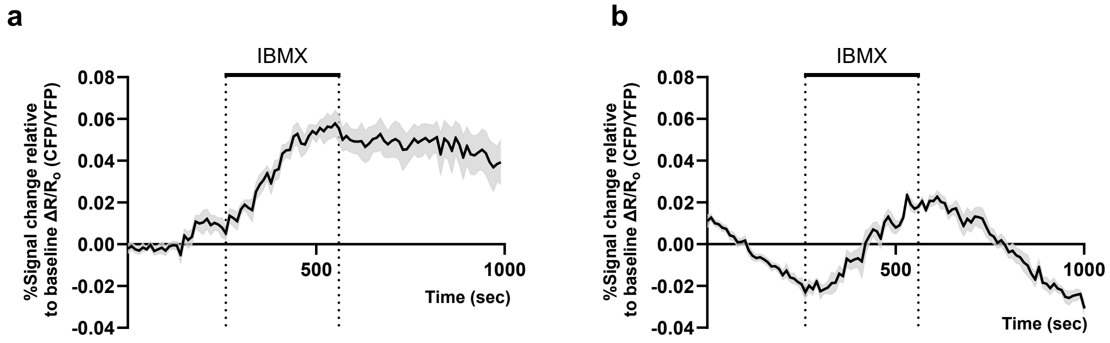

Supplemental Figure 11: Impact of unspecific PDE Inhibition on cGMP signaling dynamics in endothelial cells and podocytes. Acute kidney slices from Tie2:Cre/cGi500 mice (**a**) and Pod:Cre/cGi500 mice (**b**) were superfused (dotted lines) with 500 µM IBMX in the time period from 200 sec to 500 sec. Stimulation was followed by pre-tempered 1X KHB (37°C), a total time frame of 1000 sec was recorded. Graphs represent the mean of baseline-normalized FRET (CFP/YFP) ratios ($\Delta R/R_o$) of all analyzed glomeruli. Data represent mean ± SEM of at least 10 glomeruli obtained from slices of two Tie2:Cre/cGi500 mice and 10 glomeruli from slices of two Pod:Cre/cGi500 mice.
